# Management of Retinoblastoma: A Challenge in Pediatric Ophthalmology

**DOI:** 10.3390/life16040572

**Published:** 2026-04-01

**Authors:** Claudia Carolina Cruz-Gálvez, Víctor Manuel Villar-Calvo, Juan Carlos Ordaz-Favila, Martha Edith Cancino-Marentes, Ximena García-Vicera, Tannia Isabel Campos-Bayardo, Vanessa Bosch-Canto

**Affiliations:** 1 Physiology Department, Centro Universitario de Ciencias de la Salud, Universidad de Guadalajara, Guadalajara 44348, Mexico; 2 Pediatric Ophthalmology, San Javier Hospital, Guadalajara 44670, Mexico; 3 Pediatric Ophthalmology, Instituto Nacional de Pediatría, Universidad Autónoma de México, Ciudad de México 04530, Mexico; 4 Master of Public Health, Academic Unit of Medicine, Universidad Autónoma de Nayarit, Tepic 63000, Mexico; 5 Pediatric Oncology, Instituto Nacional de Pediatría, Universidad Autónoma de México, Ciudad de México 04530, Mexico

**Keywords:** retinoblastoma, intraocular tumor, children, ocular oncology, pediatric ophthalmology

## Abstract

Retinoblastoma (Rb) is the most common intraocular malignancy. If left untreated, Rb can result in death within 1–2 years. However, with timely detection and proper treatment, the survival rate is as high as 98%. The primary goal of Rb treatment is to eliminate cancer and save the patient’s life, while the second goal includes preserving the eye and vision. Management of Rb requires timely detection, differentiation of the tumor from similar conditions, staging, making informed decisions about the best therapeutic approach, and close follow-up to detect any signs of tumor recurrence. There are several treatment options available for Rb. Early detection and proper treatment are essential in saving the lives and vision of children affected by Rb. Progress in developing efficient diagnostic and therapeutic techniques brings hope to patients with Rb and their families. The PubMed database was utilized to identify relevant references published during the last 35 years. This article shows basic and current concepts on managing Rb, which encompasses diagnosis, evaluation, treatment, follow-up, and challenges.

## 1. Introduction

**Figure 1:**
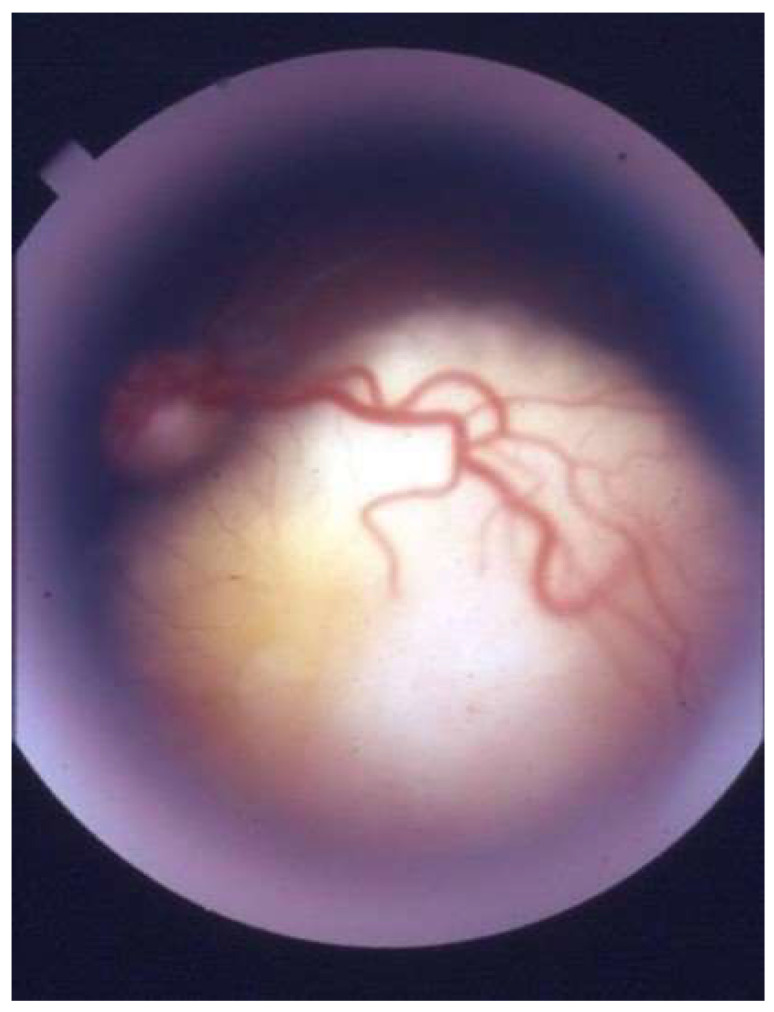
Retinoblastoma.

Retinoblastoma (Rb), named by Verhoeff, is the most common intraocular malignancy (Figure 1) [1,2]. The American Ophthalmological Society adopted the Rb term in 1926 [1]. Rb is caused by mutations in the retinoblastoma gene (*RB1*) [1,3,4,5]. Autosomal dominant inheritance is observed in 30–40% of cases, while the remaining cases are of the non-inherited sporadic type [4]. Leukocoria is the most common sign of Rb, followed by strabismus [1]. The most frequent differential diagnoses of Rb include Coats disease, persistent fetal vasculature, vitreous hemorrhage, familial exudative vitreoretinopathy, and rhegmatogenous retinal detachment [1]. Rb, if left untreated, can result in death within 1–2 years [6]. However, with timely detection and proper treatment, the survival rate can be as high as 98% [2,7]. It is noteworthy that in Low-Income Countries (LIC), the survival rate is considerably lower, only 40%, due to the limitations of healthcare [2,8]. Therefore, early detection and the correct staging (Table 1) of Rb is essential because it allows for more accessible, less expensive treatments and the best prognosis.

**Figure 2:**
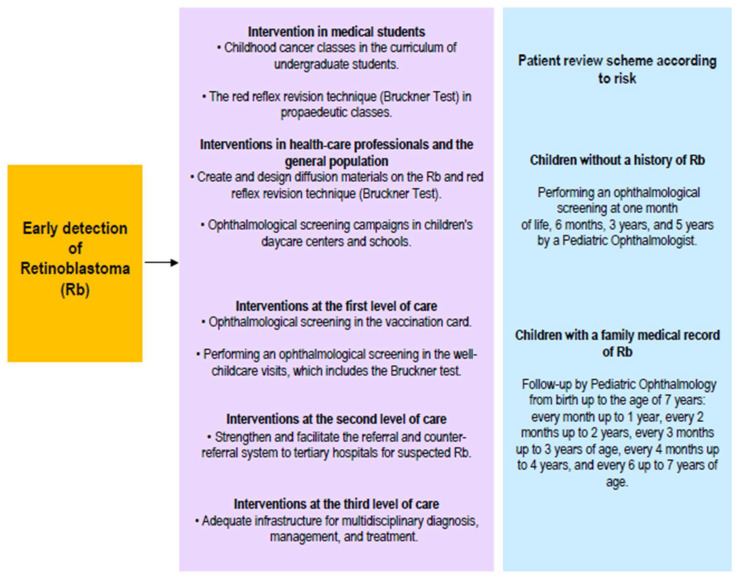
Strategies for achieving early detection of retinoblastoma.

**Figure 3:**
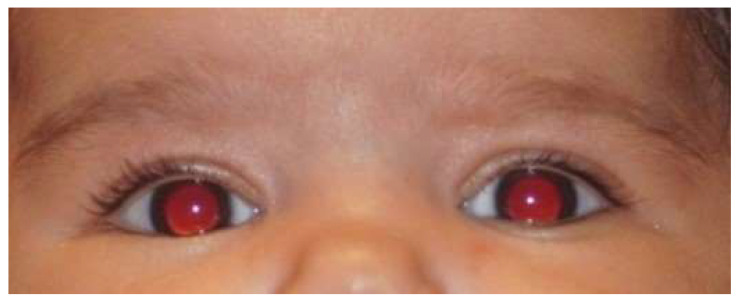
The Bruckner test (red reflex) is normal in both eyes.

**Figure 4:**
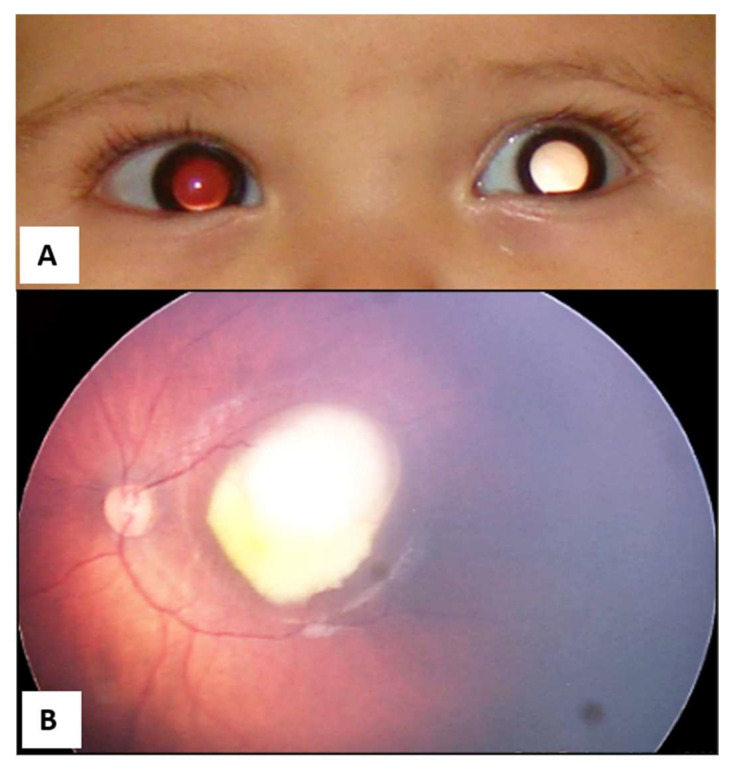
(**A**) Leukocoria in the left eye secondary to retinoblastoma. (**B**) Confirmation of Group C retinoblastoma in the left eye.

Information campaigns on Rb targeting the general population, nursing or medical students, pediatricians, and healthcare professionals can help achieve this (Figure 2) [5]. Rapid Rb detection tests, such as the Bruckner test, also contribute to early detection. The Bruckner test involves simultaneously evaluating the red reflex of both eyes using a direct ophthalmoscope (Figure 3) [9]. Performing an ophthalmological screening in the well-childcare visits, which includes the Bruckner test, is an easy, fast, and simple strategy that could generate substantial benefits in the early detection of Rb when leukocoria is evidenced (Figure 4), and even of other eye disorders or pathologies in children [9].

**Figure 5:**
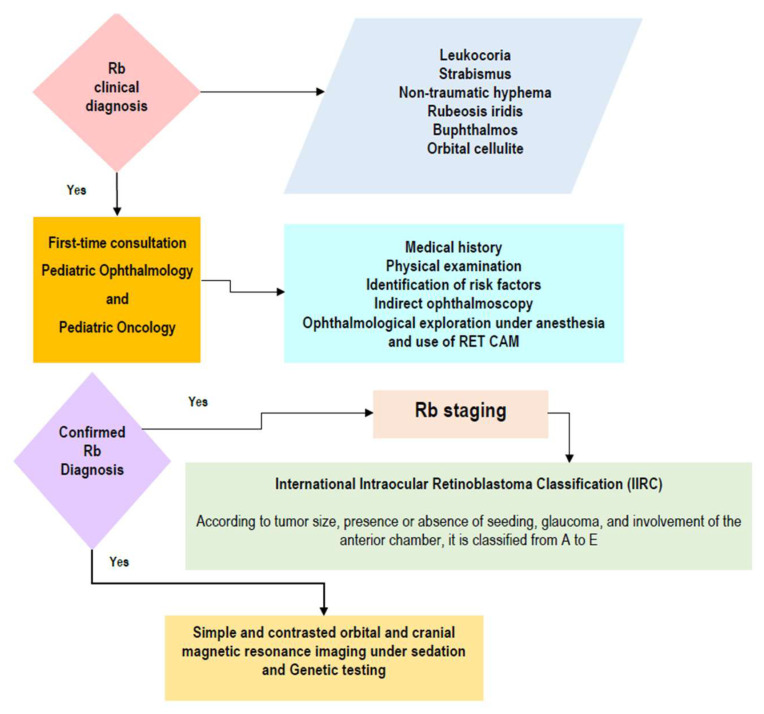
The initial approach to suspected retinoblastoma.

The initial approach to suspected Rb is essential for a better prognosis (Figure 5). The clinical presentation of Rb is widely recognized. In 60% of cases, leukocoria is the initial sign, which can be identified through the Bruckner test. Unfortunately, this sign is often missed by pediatricians, ophthalmologists, or general practitioners [5].

Managing Rb requires timely detection, differentiation of the tumor from similar conditions, staging, making informed decisions about the best therapeutic approach, and close follow-up to detect any signs of tumor recurrence [6]. It is a complex process that requires a lot of attention and care, but it is critical for ensuring the best possible outcomes for patients [6].

**Figure 6:**
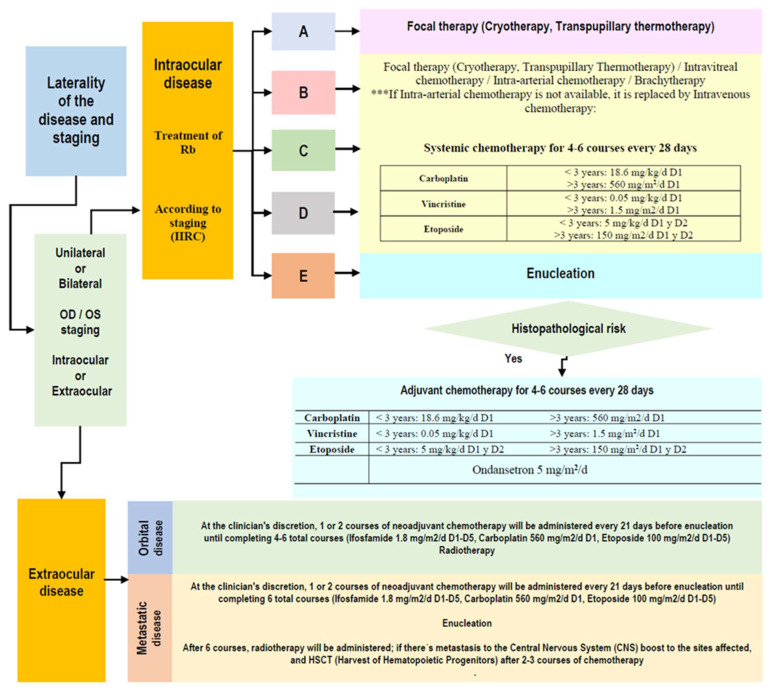
Algorithm for retinoblastoma treatment.

The primary goal of Rb treatment is to eliminate cancer and save the patient’s life, while the second goal includes preserving the eye and vision [6,10]. Management of intraocular Rb includes chemotherapy and focal treatments such as Transpupillary Thermotherapy (TTT), Cryotherapy (Cryo), and Brachytherapy. Enucleation and External Beam RadioTherapy (EBRT) are also treatment options [11,12]. Chemotherapy treatment for Rb includes Intravenous Chemotherapy (IVC) and local chemotherapy delivered by intracameral, intravitreal, and intra-arterial routes. If a patient is diagnosed with extraocular or metastatic Rb, specialized protocols are implemented to safeguard the life of the patient [13] (Figure 6).

This review focuses on the management of Rb and its challenges. The PubMed database was utilized to identify relevant references published during the last 35 years. This article shows basic and current concepts on managing Rb, which encompasses diagnosis, evaluation, treatment, follow-up, and its challenges. This review seeks to encompass the essential aspects of Rb that allow an updated understanding of this pathology. This review aims to show, in a practical, summarized, and updated way, the essential aspects of Rb that allow the reader sufficient knowledge of the fundamental aspects of Rb.

## 2. Retinoblastoma Treatment

During this century, treatment for Rb has significantly improved. Advanced techniques have opened up new possibilities for preserving the eye and its visual function more effectively.

At the beginning of the 20th century, significant advancements were achieved through IVC for treating Rb, allowing for intraocular tumor reduction, the treatment of micrometastases, and a remarkable increase in the survival and eye-salvage rates of patients with Rb [5]. Nevertheless, IVC carries the risk of severe systemic adverse reactions: Carboplatin is known to cause ototoxicity, and Etoposide has the potential to cause leukemia [2,5,14].

Over the past decade, treating Rb has been revolutionized by targeted approaches such as Intra-arterial Chemotherapy (IAC) or Intravitreal Chemotherapy (IVitC). These methods have greatly improved the chances of saving the eye, particularly in advanced cases [12,15].

Therapies such as IAC and IVitC have changed the way we treat children with Rb in that the treatment is more focused; we have better intravitreal concentrations of chemotherapy medications with fewer side effects and better outcomes [16]. Hence, these are more effective and less toxic than conventional systemic chemotherapy [17]. Despite the variety of therapeutic methods, refractory or recurrent cases do occur [18].

Rb centers worldwide are transitioning from systemic to ocular-delivered chemotherapy for better success rates [10]. Nonetheless, these novel treatment techniques must be standardized at all Rb centers worldwide, especially in LIC. Even when results are good with the new therapies, it is expensive and challenging to implement IAC and Brachytherapy [10].

The management of Rb should be customized according to various factors, such as disease staging, for example, through the International Intraocular Retinoblastoma Classification (IIRC) [6] (Table 1), age, laterality, the presence of a germline mutation, and liquid biopsy results (if feasible).

Before making any treatment decisions, conducting a thorough ophthalmological evaluation [5,19] should include a bilateral dilated ocular fundus examination under general anesthesia [5,20] and high-resolution simple and contrast-enhanced Magnetic Resonance Imaging (MRI) orbital and cranial (after the initial approach, request as often as each case requires) [5,21]. Photographs of the tumor should be taken when possible. The Pediatric Oncologist should also schedule a consultation with the patient [21]. The frequency, studies, and procedures for follow-up visits will be adapted to each case.

### Treatment of Retinoblastoma According to IIRC Staging

Group A

Focal Therapy

**Figure 7:**
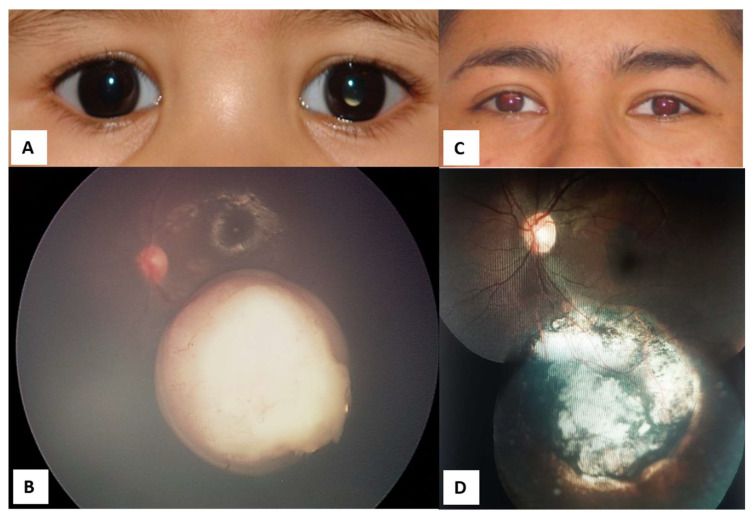
Managed retinoblastoma with intravenous chemotherapy and cryotherapy (before intra-arterial chemotherapy availability in México). (**A**) Four-month-old male patient with retinoblastoma in the left eye, pre-treatment. (**B**) Confirmation of Group C retinoblastoma in the left eye. (**C**) The same patient, 17 years after treatment. (**D**) Inactive retinoblastoma 17 years after treatments.

**Cryotherapy (Cryo).** The treatment consists of a freeze-and-thaw technique [5] repeated three times under indirect ophthalmoscopic control using scleral indentation [22,23]. For peripheral lesions, the cryotherapy probe is placed on the conjunctiva, while for more posteriorly located lesions, it is placed directly on the sclera following a conjunctival incision [21]. It treats small tumors less than 3 mm and subretinal seedings. Retinal detachment has been reported with extensive cryotherapy [21]. Cryotherapy is used in combination with IVC or IAC (Figure 7) [21].

**Figure 8:**
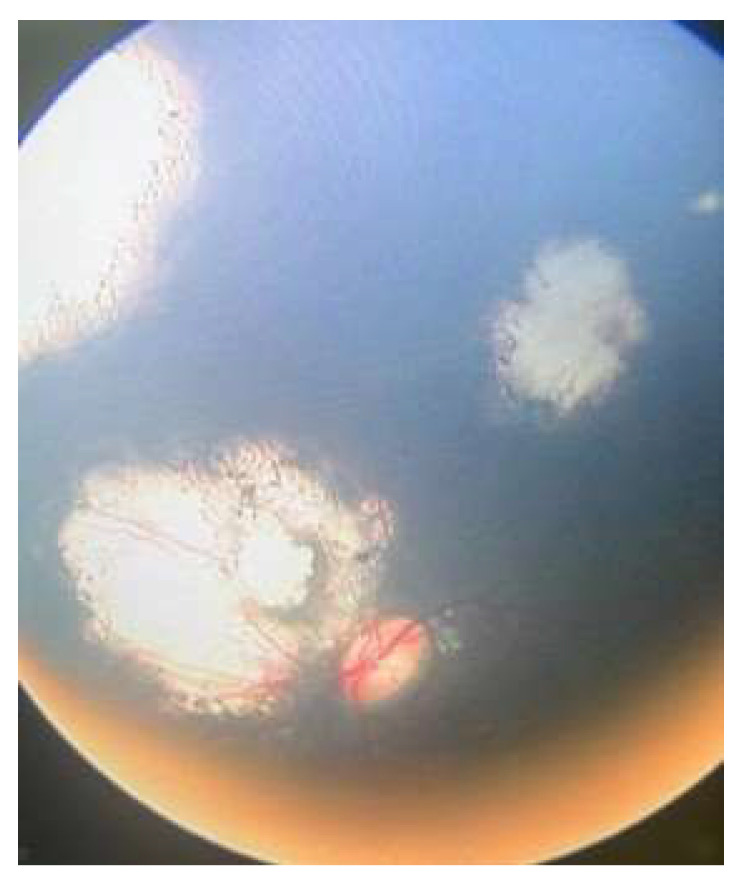
Managed retinoblastoma with transpupillary thermotherapy.

**Transpupillary ThermoTherapy (TTT).** This is the primary treatment for small tumors less than 3 mm; it can also be used in combination with IVC (Figure 8) [22]. Hyperthermia is administered using an 810 nm diode laser [22] continuously, with 30–50 s burn durations [23]. Several complications may arise from TTT, such as iris atrophy, focal cataracts, and retinal complications [21].

Groups B, C, and D.

Focal therapy.

Cryo or TTT (plus other options, including IAC, IVC, IVitC, or brachytherapy) [22].

**Figure 9:**
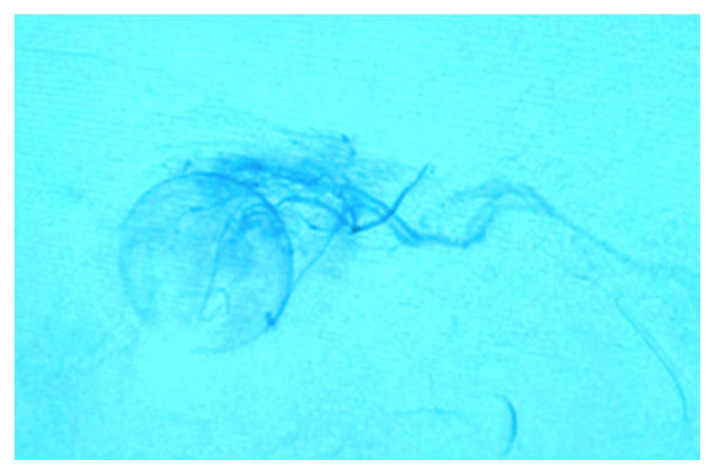
Intra-arterial chemotherapy.

**Figure 10:**
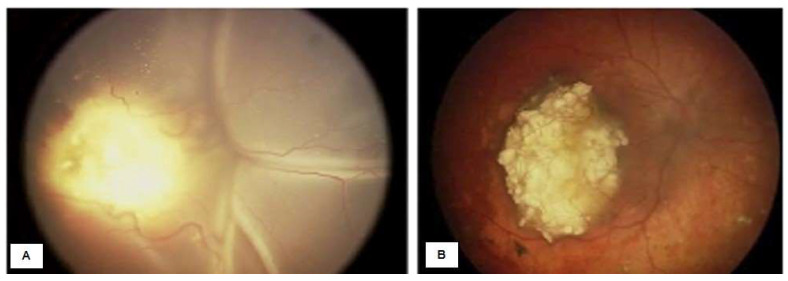
Managed Group D retinoblastoma with intra-arterial chemotherapy. (**A**) Pre-treatment; (**B**) post-treatment.

**Intra-arterial Chemotherapy (IAC).** This is the primary cornerstone treatment for Rb at present. The Kaneko drug-delivery method through the intra-arterial route enables direct delivery into the ophthalmic artery, limiting the systemic distribution of chemotherapy and its adverse effects [5,14]. The IAC technique was popularized by Abramson et al. [2]. The procedure starts with a microcatheter in the femoral artery that flows into the ophthalmic artery guided by fluoroscopy (Figure 9 and Figure 10). The chemotherapy drugs used in IAC are Melphalan, Topotecan, and Carboplatin [24]. One or a combination of these drugs can be employed monthly for two or three sessions [10,25,26]. IAC has proven effective for treating Rb in both unilateral and bilateral cases, even in infants as young as 3 months of age [27]. When administering chemotherapeutic drugs through IAC directly into the tumor, the concentration in the intraocular lesions is 10 times higher than when administered intravenously [14]. IAC treats advanced Rb that could not be treated with systemic chemotherapy [28]. However, it is a complex procedure that could be expensive for LIC. It must be performed in a Hemodynamics suite adapted for angiography and by an experienced interventional neuroradiologist. Ophthalmic vascular events can occur with this treatment [21].

**Figure 11:**
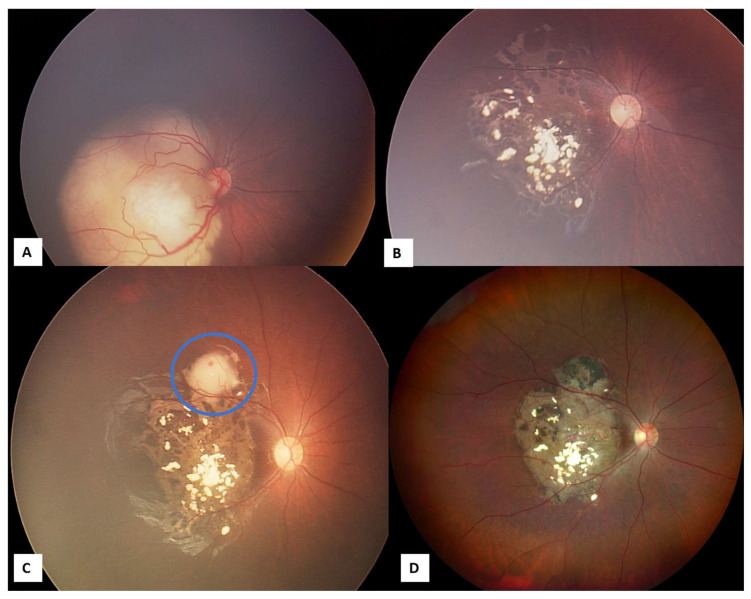
Managed retinoblastoma with intravenous chemotherapy, cryotherapy, and transpupillary thermotherapy TTT (before intra-arterial chemotherapy availability in México). (**A**) Eight-month-old female patient with Group C retinoblastoma in the right eye, previous intravenous chemotherapy and cryotherapy. (**B**) Inactive retinoblastoma. (**C**) Tumor activity (Blue circle) that was managed with TTT. (**D**) Inactive retinoblastoma 12 years after treatments.

**Intravenous chemotherapy (IVC).** IVC was introduced during the 1990s [6]. The most frequently used agents include Carboplatin, Etoposide, and Vincristine (VEC), administered monthly [6,29]. The consecutive cycles range in number between 6 and 9 [26]. IVC continues to be employed globally to control intraocular Rb (Groups B and C) and prevent systemic metastasis [6,29]. IVC is used alone or combined with focal therapies, such as TTT, Cryo, and brachytherapy (Figure 11) [6]. IVC is indicated in bilateral cases, confirmed germline mutation, in patients with suspected optic-nerve invasion, or in tiny infants waiting to gain weight for IAC [16,21]. The adverse effects of IVC include nausea, vomiting, transient alopecia, cytopenia, and fever [21].

**Figure 12:**
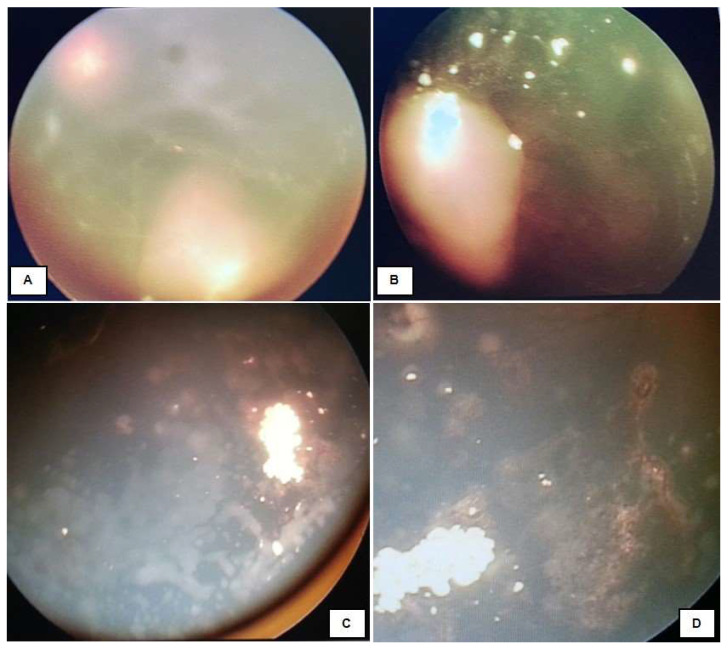
Managed Group D retinoblastomas with intravitreal chemotherapy. (**A**) Pre-treatment; (**B**–**D**) post-treatment.

**Intravitreal Chemotherapy (IVitC)**. Intravitreal administration of chemotherapy is an effective treatment for Rb. Several teams have described this treatment, which was formalized by Munier et al. [5]. IVitC delivers the highest drug concentration into the vitreous while minimizing systemic drug concentration [12]. IVitC is indicated for cases where vitreous seeds persist or recur despite other treatments (Figure 12) [12,21]. The drug regimen for IVitC consists of Melphalan and Topotecan, either used alone or in combination. To effectively control vitreous seeds without harmful side effects, 20–30 μg doses are recommended to be administered every 2–4 weeks [5,30,31]. Topotecan is also used in doses of 90 μg. IvitC may lead to ocular adverse events such as cataracts or vitreous and sub-retinal hemorrhage [21].

**Brachytherapy**. Brachytherapy for Rb was pioneered by Moore et al. and was further defined by Stallard, Sealy, Lommatzsch, and Vollmar [32]. Brachytherapy is a form of radiotherapy wherein the irradiation source is placed adjacent to or near the target tissue or organ [22]. Iodine and ruthenium plaques are currently the most frequently used plaques [32]. Brachytherapy is a secondary treatment for medium-sized tumors that do not respond to chemotherapy and that have localized vitreous or sub-retinal seeding [21]. It is typically utilized after recurrence following IVC or IAC [33]. The ocular adverse events associated with brachytherapy are cataracts and radiation-associated ophthalmic events.

Group E

**Enucleation.** This option continues to be used in advanced cases. Current indications could be tumor progression despite conventional treatment, active Rb without vision, unilateral Group E, optical-nerve involvement, and orbital stage [34,35,36,37]. The globe must undergo a detailed histopathological examination after enucleation. This report will provide us with important information concerning tumor extension. Pathologic features found in enucleated eyes indicate that the risk of metastases is as follows: post-laminar optic-nerve invasion; massive choroidal invasion > 3 mm; anterior chamber invasion involving the iris or ciliary body, and scleral or extra-scleral involvement. However, the definition of the features is widely debated [21,38,39]. If one of these risks is present, the pediatric oncologist should provide adjuvant IVC to prevent metastases. Parents should be correctly informed on the risk of metastases by conserving a nonfunctional eye with intensive eye-conserving treatment. It is necessary to achieve aesthetic rehabilitation.

**Treatment for extraocular tumor extension and metastasis prevention.**

**External Beam Radiotherapy**

**Figure 13:**
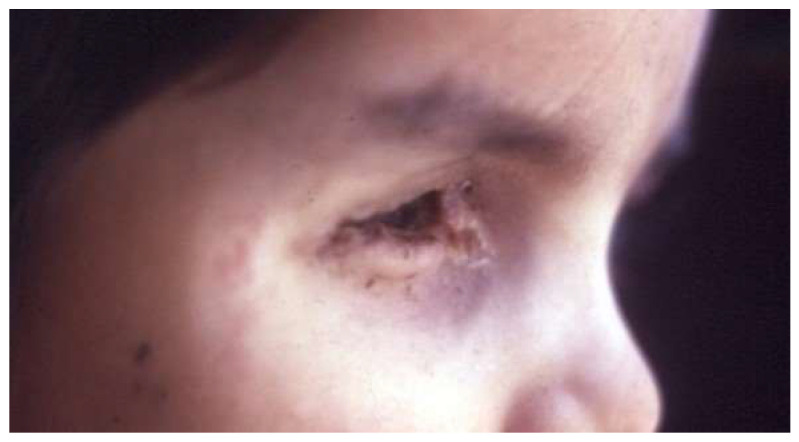
After-effects of external beam radiotherapy.

External Beam Radiotherapy (EBRT) is rarely used at present. In cases of extraocular tumor extension, orbital recurrence, and positive optic-nerve margin following enucleation, EBRT remains a viable treatment option in conjunction with IVC [21,40]. EBRT causes osseous-growth retardation, possibly leading to facial deformity (Figure 13) [22,41,42,43]. The most severe risk associated with EBRT is the high possibility of developing second primary tumors [22]. The incidence of a second cancer inside and outside of the irradiated field has been estimated to be 51% at 50 years of age [5]. The most commonly observed subsequent malignant neoplasms (SMN) are sarcomas, melanomas, and myelogenous leukemia [2,44].

## 3. Metastases

In Rb, the presence of metastases is devastating. In countries with access to advanced healthcare, the likelihood of metastasis in children with Rb is less than 10%, but this percentage is higher in LIC [2]. According to Kaliki et al., patients with non-high-risk Rb did not experience metastasis; however, 4% of high-risk patients died due to metastasis [45,46]. The chance of survival for patients with distant metastasis was nearly zero, employing high-dose chemotherapy and autologous stem-cell transplantation [47].

## 4. Retinoblastoma Characterization for Diagnosis, Prognosis, Predictive Information, and Treatment Choice

**Liquid Biopsy**

Aqueous humor (AH) liquid biopsy allows the in vivo detection of tumor-derived DNA in patients with Rb [48]. The AH liquid biopsy provides a wealth of genetic information specific to Rb [48]. In 2017, it was shown that the AH liquid biopsy, attained via paracentesis, comprises a robust source of tumor-derived cell-free DNA (cfDNA) [48,49,50,51]. cfDNA can be effectively isolated to detect relevant molecular biomarkers in eyes actively undergoing treatment or before treatment. Tumor DNA present in the AH of eyes with Rb can be utilized to identify the Rb gene (*RB1*), single-nucleotide variants (SNV), somatic copy number alterations (SCNA), and tumor methylation status to estimate tumor fraction, metabolomic signatures, and the expression of secreted peptides [48]. Liquid biopsy of aqueous humor at the time of diagnosis can provide the highest concentration of nucleic acids and proteins and may prove to be most useful for *RB1* mutational testing. Even during therapy, aqueous humor biopsy can yield sufficient DNA concentrations for low-pass whole-genome sequencing and SCNA detection, which can aid in predicting prognosis and monitoring trends in tumor fraction that are associated with ocular outcomes [49]. The AH liquid biopsy in Rb could pave the way for a better understanding of mechanisms for treatment response, resistance, and recurrence in Rb and could provide specific therapeutic targets to improve globe salvage [51,52].

**Genetic Counseling**

Genetic counseling in Rb represents a challenge for the Treating Physician [53]. Rb genetics supports appropriate management in the clinical setting through correct information and the genetic testing of patients and families [54]. It explains the genetics of Rb appropriately and in simple language that can aid parents and patients in their adulthood in terms of their proper understanding of the disease [55]. Individuals with inherited Rb are more likely to develop ocular and non-ocular tumors [50]. Additionally, transmitting the mutated gene to their future offspring is possible [50]. Patients with heritable Rb have an increased risk of SMN [2]. It is appropriate to offer genetic counseling (including the discussion of potential risks to offspring and reproductive options) to young adults affected with or at risk of Rb [56]; in vitro fertilization with a pre-implantation diagnosis can be discussed in future family planning, in addition to pointing out the importance of a timely pediatric ophthalmologic examination from birth up to age 7 years, [57] for the future descendants of Rb survivors. Rb survivors should undergo lifelong monitoring, particularly those with a germline mutation.

**Radiomics Model**

Radiomics uses the high-throughput extraction of advanced quantitative features to objectively and quantitatively describe tumor phenotypes [58]. These quantitative features can provide a valuable oncological diagnosis, prognosis, or predictive information [58]. Magnetic resonance imaging (MRI) is a critical technique for detecting the presence of post-laminar optic-nerve invasion (PLONI) in patients with Rb [59,60,61,62]. PLONI is a risk factor for developing metastatic disease [62]. The MRI-based radiomics model to predict PLONI in patients with Rb was shown to be superior to visual assessment and may serve as a potential tool to guide personalized treatment [58].

**Artificial Intelligence**

Artificial Intelligence (AI) in medicine aids in developing treatment algorithms and predictive models via electronic medical records and Big Data, or softbots [63]. In the field of ophthalmology, AI has been capable of analyzing data and images, thus aiding in the diagnosis and staging of various ocular pathologies, including strabismus, refractive error, keratitis, keratoconus, cataracts, glaucoma, optic-disk abnormalities, diabetic retinopathy, age-related macular degeneration, or the retinopathy of prematurity [63,64,65,66,67]. AI has also been employed to develop treatment algorithms and to prognosticate ocular pathologies. In ophthalmology, AI entertains the potential for universalizing patient access to disease screening and diagnosis, for expediting treatment as appropriate, and for monitoring outcomes [67]. AI is a promising assessment tool for Rb [63]. Using an AI model, it is possible to screen children in the community for Rb by utilizing fundus images captured on an inexpensive nonmydriatic fundus camera. This approach can help diagnose the condition at an early stage [63].

**Genomics**

The majority of Rb cases result from biallelic *RB1* gene inactivation, although a small subset (1–2%) is initiated through *MYCN*-gene amplification in the absence of *RB1*-gene inactivation [68,69,70,71]. Other genetic modifications have also been associated with Rb. Genetic modifiers, such as *MDM2*, *MDM4*, or *MED4*, and polymorphisms in *p53*, *CDKN1A*, *CDKN2A*, and *BCOR* could also influence hereditary Rb development [70]. These findings have diagnostic, clinical, and response-to-treatment implications.

*MYCN* amplifications were recently identified in a subset of aggressive Rb in infants [72] with relative resistance to typical therapeutic approaches to Rb [73]. *MYCN*-amplified Rb possesses distinct features compared with the *RB1* pathogenic variant on MRI, including tumors in a peripheral (anterior) location with a plaque or pleomorphic shape, irregular margins, tumor–retinal folding, and peri-tumor blood [72]. miR-142-5p restricted cell proliferation, migration, invasion, and enhanced cell apoptosis in Rb cells. *MYCN* is adversely controlled by miR-142-5p [74]. *MYCN* upregulated m6A reader functions to promote Rb cell proliferation and tumor growth [75]. Another mutated gene in Rb (7–13% of cases) is the epigenetic modifier gene *BCOR* [76].

Investigations propose *FGFR4*, *NQO1*, *ACADS*, *CX3CR1*, *GBE1*, *KRT85*, and *TYR* as possible candidate genes involved in Rb oncogenesis. A significant proportion of sporadic Rb exhibits somatic mosaicism for *RB1* mutations [70]. Early onset Rb tumor cells express an immune gene-expression signature followed by the accumulation of dendritic, monocyte, macrophage, and T-lymphocyte cells in the Rb tumors [77]. These findings represent potential effective targets for Rb treatment.

## 5. Retinoblastoma Challenges

Rb management requires a highly trained and up-to-date multidisciplinary team comprising a pediatrician, pediatric ophthalmologist, pediatric oncologist, pediatric geneticist, pediatric radiologist, and pediatric psychologist, which can be complex to integrate, especially in developing countries [21]. The prognosis of Rb is excellent if it is diagnosed early and treated appropriately, with cure rates higher than 90% [78]. Regrettably, the epidemiology of Rb reveals inequities in access to timely diagnosis and treatment between developed countries and LIC [78,79,80]. Many patients in LIC frequently present in an advanced Rb stage due to inadequate knowledge of the early signs of Rb, limited medical-care resources, and postponed referral [15,78]. This leads to a mortality rate that is significantly higher in LIC. In Asia and Africa, 40–70% of children with Rb die, while in Europe, Canada, and the U.S., the mortality rate is only 3–5% [60].

Early detection of Rb is fundamental; therefore, fast and easy detection strategies such as the Bruckner test contribute to the effective management of this disease. Rb can be detected by means of the Bruckner test, exhibiting in a patient a shining white pupil, or “Leukocoria” [9,81]. OCT (Optical Coherence Tomography) can aid in identifying very small Rb that are not detectable (that are “invisible”) by indirect ophthalmoscopy [82,83,84], and the latter should be more widely used for the timely detection of Rb. Teleophthalmology and tele-education resources have been validated in diverse ocular pathologies. In Rb, there is the option of online mentorship via Orbis Cybersight, which has shown encouraging results [85]. Early detection and proper treatment are essential in saving the lives and vision of children affected by Rb.

Today, there are several forms of Rb treatment, including using chemotherapy through different routes of administration, focal therapy, EBRT, and enucleation. Unfortunately, none of these options is foolproof [78].

IVC represents an essential part of Rb treatment, proving to be effective not only in controlling the disease but also in preventing metastasis and reducing the occurrence of second cancers in the long term [86]. Although the potential risk of IVC toxicity [87], the lower concentration of IVC achieved at the intraocular level secondary to the blood–retinal barrier, and the chemoresistance of Rb should not be ignored [9,88].

Carboplatin is one of the most important drugs in the standard chemotherapy regimen for Rb. Still, it activates the transcription factor nuclear factor-kappa B (NF-ĸB), which entails Rb cell survival [9]. Therefore, it is important to look for additional drug options that are effective per se for Rb or that enhance the effect of chemotherapy without increasing its dose. Molecularly targeted drugs may be more effective and less toxic [89].

The management of Rb will likely be substantially modified based on the more frequent use of liquid biopsy [48], molecular biomarkers [79], updated genetic testing [80], genotype–phenotype tumor correlations [90], prenatal diagnosis [91], Rb subtypes [92], and new technologies such as radiomics models and AI.

The investigation of Rb emerging treatments includes new or repositioned drugs (alone or in combination), immunotherapy, novel delivery systems, oncolytic adenovirus, gene therapy, and molecularly targeted therapies [78,86,93,94].

New intracellular targets can potentially lead to the identification of novel Rb drugs. Examples of this are Bcl-2 proteins, bromodomain and extra-terminal motif proteins (BET), MDM2/MDM4 inhibitors, NF-kB, histone deacetylase, kinesin spindle protein, STAT3, cyclin-dependent kinase (CDK) 4 and 6, p-53, MYC, and GABA receptors [95].

In vitro and in vivo studies showed synergistic inhibitory effects of the combination of nutlin-3a and topotecan. miR-129 could be a novel targeted therapy for Rb treatment. Bortezomib in vivo was mediated through the induction of apoptosis in 2 Rb cell lines by blocking the NF-κB [96].

The use of nanotechnology offers the possibility of new therapeutic strategies in Rb [97]. Organic polymers or inorganic nanoparticles loaded with synthetic or natural drugs have been developed. All systems showed an increase in the bioavailability of the drug and an ability to pass the blood–retinal barrier and reduce the side effects of anticancer conventional drugs [96].

Epigenetic markers (noncoding RNAs, DNA methylations, RNA modifications, chromatin conformations, and histone modifications) play a vital role in the inactivation of *RB1*. This knowledge could contribute to future Rb treatment strategies or as diagnostic biomarkers [98,99,100].

Fully understanding Rb tumorigenesis is still an ongoing process. The creation of organoid models is a strategy that enables us to understand this process in greater detail [101,102,103].

The proposal to subdivide the Rb group E could open up a new possibility for management in this group [104,105].

Recently, in Rb patients with unilateral pathological high-risk features, undergoing frontline enucleation, the administration of three cycles versus six cycles of adjuvant chemotherapy was studied, resulting in a non-inferiority of three cycles versus six cycles [106]. This results in less toxicity, less decline in quality of life, and lower treatment costs. However, it should be noted that observations have been made on this research, for example, that different types of high-risk histopathological findings involve different prognoses, so this reduced treatment should not be taken as a standardized management guide; what should prevail is the individualization of each case.

IAC and IVitC have improved conservative treatment and eye salvage in many cases of Rb. For instance, the Grupo de America Latina de Oncología Pediátrica (GALOP), through a consensus document on the treatment of Rb, found that IAC was the preferred therapy in advanced disease, and IVitC was the primary treatment for vitreous seeding [107]. Also supporting part of the previous findings, Wen et al. compared IAC vs. IVC, and based on their findings, they indicate that IAC is a first-line treatment in children with advanced unilateral Rb [108]. IAC has a high success rate that has improved over the past 15 years [109]. However, IAC and IVitC imply a more significant health expenditure to make available the necessary resources (drugs, hospitalization, hospital infrastructure, and trained personnel) [110,111,112,113,114,115].

However, even with the challenges posed by IVitC, it continues to demonstrate its importance as a therapeutic strategy in Rb. An example of this is that high-dose IVitC topotecan (90 μg), in conjunction with other therapies, has proven to be an option for recurrent Rb, which is difficult to handle [116,117].

Unfortunately, systemic metastases are the cause of significant Rb mortality in LIC, which evidences the disparity in the management of Rb [118].

Rb survivors, particularly those with hereditary Rb or those exposed to radiotherapy, are at risk of developing second primary tumors [119,120,121]; therefore, they require lifelong follow-up, which entails costs, whether at a personal or institutional level. According to Zhao et al., the second primary tumors in Rb survivors are more common in the fourth decade of life [119]. They also point out that 25% of the Rb survivors with second primary tumors in their research had a history of unilateral Rb, and approximately 25% of Rb survivors analyzed were treated with surgery alone and not with chemotherapy or radiotherapy, and yet developed second primary cancers [119].

Cases outside the usual presentation of Rb also present a challenge. Rb is rare after 5 years of age. Two mechanisms associated with this phenomenon have been described: the novo Rb and as a sequela of oncogenetic mutations in a pre-existing retinocytoma or a retinoma. There are reported cases of patients aged 14 years old or even adults [122,123]. Outside of the usual presentation of Rb, reaching a diagnosis can be more complex.

Additional comorbidities such as tuberculosis or any other infection, diseases that may require a prolonged period of recovery, human immunodeficiency virus (HIV)/acquired immune deficiency syndrome (AIDS), and malnutrition need to be taken into account in the management of unilateral Rb, for example, a reduction in chemotherapy doses of the first cycle of chemotherapy is recommended [124].

Another important aspect to consider in Rb is the psychological impact of the diagnosis on both the family and the patient, particularly due to the impact of enucleation, which can lead to an altered body image, impaired binocular vision, and even the costs associated with the need for an ocular prosthesis [125].

Even if the eyeball is saved, it is important to consider that vision in that eye could also be compromised, whether due to the underlying diagnosis or the consequences of the different treatment modalities [125]. Therefore, psychological support is important from the moment the Rb diagnosis is confirmed.

The progress in developing efficient diagnostic, staging (e.g., according to IIRC or American Joint Committee on Cancer, AJJC) (Table 1, Table 2, Table 3, Table 4, Table 5, Table 6 and Table 7), [6,126] and therapeutic strategies brings hope to patients with Rb and their families. However, it also challenges healthcare systems with respect to ensuring that every patient can access early diagnosis and timely treatment [109]. WHO Global Initiative for Childhood Cancer (CURE-ALL) seeks to respond to these needs since it has as its pillars: centers of excellence and care networks, universal health coverage, regimens for management, evaluation, and follow-up [118]. Hopefully, more initiatives like this one will be developed to reduce the differences in management and prognosis between the different scenarios in which Rb can occur.

## 6. Conclusions

There are several challenges when talking about Rb. Cancer is the leading cause of death worldwide, with its incidence rising steadily [127]. Rb is not the exception [128,129,130].

Retinoblastoma survival disparities remain between developed and developing countries. In the developing countries, there are more cases and worse prognosis [127].

Although there are several therapeutic options for Rb, none of them are infallible. The priority in Rb is to safeguard the patient’s life, and this premise should not be lost sight of. Early diagnosis that allows for less complex treatment strategies is a cornerstone in the management of Rb [131].

## Figures and Tables

**Table 1:** The International Intraocular Retinoblastoma Classification (IIRC).

IIRC
**Group A** Small tumors, 3 mm or smaller in their greatest dimension, confined to the retina and located >3 mm from the fovea and 1.5 mm from the optic disk.
**Group B** Tumors greater than 3 mm, located 3 mm or less from the fovea and less than 1.5 mm from the optic disk, or that present subretinal fluid whose diameter is less than 3 mm from the margin of the tumor.
**Group C** Retinoblastoma with seeding, which can be subretinal within 3 mm of the primary tumor, or vitreous seeding located <3 mm from the primary tumor, or both vitreous and subretinal seeding <3 mm from the primary tumor.
**Group D** Retinoblastoma with diffuse seeding that may be subretinal >3 mm from the retinoblastoma, vitreous seeding >3 mm from the retinoblastoma, or a combination of both.
**Group E** Extensive retinoblastoma, which occupies more than 50% of the eye socket. It can be accompanied by neovascular glaucoma, phthisis bulbi, and/or opaque media due to hemorrhage from the anterior chamber, the vitreous, or the subretinal space. Tumors with post-laminar invasion of the optic nerve, choroid, sclera, orbit, or anterior chamber also enter this section.

**Table 2:** Definition of Clinical Primary Tumor (cT) for retinoblastoma.

cT Category		cT Criteria
**cTX**		Unknown evidence of an intraocular tumor
**cT0**		No evidence of an intraocular tumor
**cT1**		Intraretinal tumor(s) with subretinal fluid ≤5 mm from the base of any tumor
	cT1a	Tumors ≤3 mm and further than 1.5 mm from the disk and fovea
	cT1b	Tumors >3 mm or closer than 1.5 mm from the disk or fovea
**cT2**		Intraocular tumor(s) with retinal detachment, vitreous seeding, or subretinal seeding
	cT2a	Subretinal fluid >5 mm from the base of any tumor
	cT2b	Vitreous seeding and/or subretinal seeding
**cT3**		Advanced intraocular tumor(s)
	cT3a	Phthisis or pre-phthisis bulbi
	cT3b	Tumor invasion of the choroid, pars plana, ciliary body, lens, zonules, iris, or anterior chamber
	cT3c	Raised intraocular pressure with neovascularization and/or buphthalmos
	cT3d	Hyphema and/or massive vitreous hemorrhage
	cT3e	Aseptic orbital cellulitis
**cT4**		Extraocular tumor(s) involving the orbit, including the optic nerve
	cT4a	Radiologic evidence of retrobulbar optic nerve involvement, thickening of the optic nerve, or involvement of orbital tissues
	cT4b	Extraocular tumor is clinically evident with proptosis and/or an orbital mass

From: Amin MB, Edge SB, Greene FL et al., eds.: *AJCC Cancer Staging Manual*. 8th Ed. New York, NY: Springer, 2017, pp. 819–831. Ref. [126].

**Table 3:** Definition of Heritable Trait (H) for retinoblastoma.

H Category	H Criteria
**HX**	Unknown or insufficient evidence of a constitutional *RB1* gene variant
**H0**	Normal *RB1* alleles in blood tested with demonstrated high-sensitivity assays
**H1**	Bilateral retinoblastoma, retinoblastoma with an intracranial primitive neuroectodermal tumor (i.e., trilateral retinoblastoma), patient with a family history of retinoblastoma, or molecular definition of a constitutional *RB1* gene variant

From: Amin MB, Edge SB, Greene FL, et al., eds.: *AJCC Cancer Staging Manual*. 8th Ed. New York, NY: Springer, 2017, pp. 819–831. Ref. [126].

**Table 4:** Definition of Pathological Primary Tumor (pT) for retinoblastoma.

pT Category		pT Criteria
**pTX**		Unknown evidence of an intraocular tumor
**pT0**		No evidence of an intraocular tumor
**pT1**		Intraocular tumor(s) without any local invasion, focal choroidal invasion, or pre- or intralaminar involvement of the optic nerve head
**pT2**		Intraocular tumor(s) with local invasion
	pT2a	Concomitant focal choroidal invasion and pre- or intralaminar involvement of the optic nerve head
	pT2b	Tumor invasion of the stroma of the iris and/or trabecular meshwork and/or Schlemm’s canal
**pT3**		Intraocular tumor(s) with significant local invasion
	pT3a	Massive choroidal invasion (>3 mm in largest diameter, or multiple foci of focal choroidal involvement totalling >3 mm, or any full-thickness choroidal involvement)
	pT3b	Retrolaminar invasion of the optic nerve head, not involving the transected end of the optic nerve
	pT3c	Any partial-thickness involvement of the sclera within the inner two-thirds
	pT3d	Full-thickness invasion into the outer third of the sclera and/or invasion into or around the emissary channels
**pT4**		Evidence of extraocular tumor: tumor at the transected end of the optic nerve, tumor in the meningeal spaces around the optic nerve, full-thickness invasion of the sclera with invasion of the episclera, adjacent adipose tissue, extraocular muscle, bone, conjunctiva, or eyelids

From: Amin MB, Edge SB, Greene FL, et al., eds.: *AJCC Cancer Staging Manual*. 8th Ed. New York, NY: Springer, 2017, pp. 819–831. Ref. [126].

**Table 5:** Definition of Clinical Regional Lymph Node (cN) for retinoblastoma.

cN Category	cN Criteria
**cNX**	Regional lymph nodes cannot be assessed
**cN0**	No regional lymph node involvement
**cN1**	Evidence of preauricular, submandibular, and cervical lymph node involvement

From: Amin MB, Edge SB, Greene FL, et al., eds.: *AJCC Cancer Staging Manual*. 8th Ed. New York, NY: Springer, 2017, pp. 819–831. Ref. [126].

**Table 6:** Definition of Pathological Regional Lymph Node (pN) for retinoblastoma.

pN Category	pN Criteria
**pNX**	Regional lymph node involvement cannot be assessed
**pN0**	No lymph node involvement
**pN1**	Regional lymph node involvement

From: Amin MB, Edge SB, Greene FL, et al., eds.: *AJCC Cancer Staging Manual*. 8th Ed. New York, NY: Springer, 2017, pp. 819–831. Ref. [126].

**Table 7:** Definition of Clinical (c) and Pathological (p) Distant Metastasis (M) for retinoblastoma.

M Category		M Criteria
**cM0**		No signs or symptoms of intracranial or distant metastasis
**cM1**		Distant metastasis without microscopic confirmation
	cM1a	Tumor(s) involving any distant site (e.g., bone marrow, liver) on clinical or radiologic tests
	cM1b	Tumor involving the central nervous system on radiologic imaging (not including trilateral retinoblastoma)
**pM1**		Distant metastasis with histopathologic confirmation
	pM1a	Histopathologic confirmation of tumor at any distant site (e.g., bone marrow, liver, or other)
	pM1b	Histopathologic confirmation of tumor in the cerebrospinal fluid or central nervous system parenchyma

From: Amin MB, Edge SB, Greene FL, et al., eds.: *AJCC Cancer Staging Manual*. 8th Ed. New York, NY: Springer, 2017, pp. 819–831. Ref. [126].

## Data Availability

No new data were created or analyzed in this study.
